# Preliminary Assessment of Commercial Biofertilizers as Biocontrol Agents of Oak Wilt

**DOI:** 10.3390/microbiolres17060115

**Published:** 2026-06-10

**Authors:** Samira Islas-Valdez, Robert Rubiano, Ryan L. Peterson, Nicole Wagner

**Affiliations:** 1 Department of Agricultural Sciences, Texas State University, 601 University Dr., San Marcos, TX 78666, USA; 2 Department of Chemistry and Biochemistry, Texas State University, San Marcos, TX 78666, USA

**Keywords:** antagonistic microorganisms, antifungal activity, biological control, non-volatile metabolites, oak wilt, volatile organic compounds

## Abstract

*Bretziella fagacearum* (formerly *Ceratocystis fagacearum* (Bretz)) Hunt is a vascular pathogen responsible for oak wilt disease, which affects various oak species in North America. Once established, management options include root disruption, removal of infected wood, and fungicide application, each with variable efficacy. This is the first study to assess three commercial biofertilizers against *B. fagacearum* in vitro, using Spectrum supplemented with Pepzyme Clear (SPC), EM-1, and Power Gelatinase and Chitinase-producing Microorganism (PGCM), as no biological methods currently exist. These biofertilizers were chosen for microbes associated with improved nutrient uptake and for their potential biocontrol activity. We conducted dual-culture plate assays, volatile organic compounds (VOCs) assays, and non-volatile metabolite assays. EM-1 and PGCM exhibited the strongest antagonistic effects for dual-culture plate assays (56% and 68%, respectively) and for VOCs assays (62% and 47%, respectively). After 15 days of exposure to non-volatile metabolites, microscopic analysis revealed severe hyphal distortions from EM-1 and PGCM. These preliminary in vitro findings suggest that PGCM and EM-1 suppressed mycelial growth of *B. fagacearum* and may be used as biological control. Further field studies are needed to understand how environmental factors and soil–tree–microbe interactions can affect their efficacy against oak wilt disease.

## 1. Introduction

Oak wilt is a vascular disease that affects oak trees (*Quercus* spp.) caused by the fungus *B. fagacearum*, which is also known by other names, such as *Ceratocystis fagacearum* (Bretz) Hunt, *Endoconidiophora fagacearum* Bretz, *Chalara quercina* Henry, or *Thielaviopsis quercina* (EPPO, 2023). Red oaks are highly susceptible to this disease and often die within a few months after infection, whereas white oaks show more resistance [1]. The differences in susceptibility are attributed to the formation of tyloses in white oaks, which mostly occlude earlywood vessels. In contrast, the vessels in red oaks remain open, facilitating the spread of the pathogen [2].

The oak wilt pathogen spreads aboveground through nitidulid beetles, which spreads spores to fresh wounds or belowground via root grafts [3]. Once *B. fagacearum* establishes itself, it invades the vascular system, producing hyphae, spores, and metabolic by-products that clog xylem vessels, disrupting the transport of water and nutrients [4]. Additionally, tyloses formed by oaks in response to the infection further contribute to vessel blockage [5].

Oak wilt has been reported in 24 U.S. states and in several Canadian provinces [1,3,6,7]. The disease causes significant tree loss, reducing important ecosystem services, including runoff control, shade, carbon sequestration, wildlife habitat, food sources, and recreational benefits. Oaks also have substantial economic importance because they provide high-quality lumber for furniture and flooring, tannins, and dyes for leather and clothing [1,3,8]. Therefore, oak wilt poses a considerable economic threat to the timber industry. In regions with a high abundance of oaks, such as Texas, replacing trees lost to oak wilt is costly, averaging $150 per inch [2]. While *B. fagacearum* primarily infects oaks (*Quercus* spp.), it can also infect several varieties of apple (Malus) and members of the Fagaceae family, including chestnuts (*Castanea* spp.), chinkapins (*Castanopsis* spp.), tanoaks (*Lithocarpus* spp.), chinquapins (*Chrysolepis* spp.) [9,10]. This situation raises significant concerns for chestnut growers and timber stand managers, highlighting the urgent need for further research to develop effective mitigation strategies [11,12].

Current management practices rely on early detection [13–16] and the fungicide propiconazole, which reduces crown loss and extends tree life but does not eradicate oak wilt from the roots or cure infected trees [3,17]. Similarly, Blaedow et al. [18] found that applying propiconazole did not prevent or eliminate *B. fagacearum* from the root system after one year. This lack of effectiveness may be due to propiconazole’s inability to move into new growth each year, allowing the pathogen to spread into new growth rings. In addition to chemical treatments, research has investigated the potential of specific endophytic bacteria, such as *Bacillus pumilus*, *Pseudomonas denitrificans*, and *Erwinia herbicola*, as biological control agents for oak wilt management. Unfortunately, these bacterial strains were ineffective in this context, as they were unable to colonize the xylem and did not produce fungicidal metabolites [19]. Growing concerns about the environmental persistence of synthetic fungicides and the potential emergence of resistant pathogen populations [20] underscore the need for sustainable alternatives, such as beneficial microbes that produce biodegradable antifungal compounds.

While microbial antagonism has been documented for numerous plant pathogenic fungi, the oak wilt pathogen remains comparatively understudied in the context of commercially formulated biocontrol products and other biological management strategies [21,22]. This gap presents an opportunity for further research. Most existing oak wilt management strategies rely on sanitation, trenching, or prophylactic fungicide injection, approaches that are labor-intensive and often cost-prohibitive at landscape or forest scale [5]. Commercial biofertilizer formulations containing consortia of bacteria and fungi represent a potentially scalable intervention, as they are already produced under standardized manufacturing conditions and distributed for agricultural use. However, their antagonistic capacity against *B. fagacearum* has not been systematically evaluated under controlled laboratory conditions [19].

Biological control agents (BCAs) are effective in suppressing fungal pathogens through both direct and indirect mechanisms. They produce antifungal metabolites and cell wall-degrading enzymes, such as chitinase, cellulase, and β-1,3-glucanase and release volatile organic compounds (VOCs) that enhance their antifungal properties [23,24]. The development of effective and eco-friendly BCAs is a promising approach for managing oak wilt.

The objectives of this study were to evaluate the antifungal activity of commercial biofertilizers against *B. fagacearum* in vitro. It is hypothesized that these three commercial biofertilizers, primarily marketed for enhancing plant nutrition and soil fertility, might inhibit mycelial growth either directly (as determined by dual-plate assays) or indirectly (through the production of volatile and non-volatile compounds). Depending on the biofertilizer employed, both antagonistic effects may play a role. To the best of our knowledge, this is the first study to investigate commercial biofertilizers as biological control agents in vitro, and no established biological methods currently exist to combat *B. fagacearum*.

The three commercial products selected for evaluation were SPC, Power Gelatinase, and Chitinase-Producing Microorganisms (PGCM), and EM-1. These products were chosen due to their content of plant growth-promoting microorganisms and nutrient-solubilizing bacteria. According to manufacturers’ descriptions, they also enhance plant immunity, suppress pests and diseases, and improve soil fertility and overall plant health. These characteristics make them suitable candidates for in vitro antifungal screening.

## 2. Materials and Methods

### 2.1. Fungal Strain and Growth Conditions

*B. fagacearum*, formerly known as *Ceratocystis fagacearum* (Bretz) Hunt (ATCC 200423), was isolated from *Quercus virginiana* in Kerr, Texas. This fungal strain was purchased from the American Type Culture Collection (ATCC, Manassas, VA, USA). The culture was propagated on potato dextrose agar (PDA) at 24 °C, following ATCC protocols. After two weeks of incubation on PDA, the colonies exhibited a fluffy mycelial mat with gray to olive-green coloration and occasional tan patches, as documented in previous reports [9,25]. For long-term preservation, the strain was stored in potato dextrose broth containing 20% glycerol and maintained at −80 °C in sterile cryovials to facilitate future applications.

The decision to use a single isolate for the preliminary screening study was made because of the limited genetic variation observed within the North American population of *B. fagacearum* [10].

### 2.2. Preparation of Fungicide and Commercial Biofertilizers

Three commercial biofertilizers, designed to improve soil fertility and enhance plant nutrition, were evaluated as potential BCAs against *B. fagacearum*.

The first biofertilizer, Spectrum, was sourced from Tainio Technology & Technique, Inc. (Spokane, WA, USA). This product comprises a consortium of beneficial microorganisms, including plant growth-promoting rhizobacteria, nitrogen-fixing bacteria, and phosphorus-solubilizing bacteria. Among the species contained in this biofertilizer and number of colony forming units (CFU) per gram are *Arthrobacter globiformis* 1 × 10^5^, *Azospirillum brasilense* 1 × 10^6^, *A. lipoferum* 1 × 10^5^, *Azotobacter chroococcum* 1 × 10^5^, *A. paspali* 1 × 10^4^, *A. vinelandii* 1 × 10^5^, *Bacillus amyloliquefaciens* 1 × 10^6^, *B. atrophaeus* 1 × 10^5^, *B. licheniformis* 1 × 10^5^, *B. megaterium* 1 × 10^5^, *B. pumilus* 1 × 10^4^, *B. subtilis* 1 × 10^5^, *B. thuringiensis* 1 × 10^5^, *Brevibacillus brevis* 1 × 10^6^, *Micrococcus luteus* 1 × 10^5^, *Pseudomonas fluorescens* 1 × 10^5^, *P. putida* 1 × 10^6^, *Rhodopseudomonas palustris* 1 × 10^4^, *Rhodospirillum rubrum* 1 × 10^4^, and *Streptomyces griseus* 1 × 10^4^.

The second biofertilizer evaluated was PGCM, obtained from BSR USA (Davis, CA, USA). This formulation contains *Bacillus velezensis* 1 × 10^8^ CFU g^−1^, 10% sucrose, crab shell powder, diatomaceous earth, bone meal, and 2% water soluble nitrogen derived from animal protein hydrolysate. Additionally, the EM-1 activation kit was acquired from TeraGanix (Rusk, TX, USA). This third biofertilizer contains 1 × 10^6^ CFU mL^−1^ of the following species: *Lactobacillus casei*, *Lactobacillus delbrueckii*, *Lactobacillus fermentum*, *Lactobacillus plantarum*, *Bacillus subtilis*, *Saccharomyces cerevisiae*, and *Rhodopseudomonas palustri*.

Propiconazole at 14.3% was sourced from Quali-Pro^®^ (Pasadena, TX, USA) and served as a positive control or fungicide. The fungicide solution was prepared according to the manufacturer’s recommended rate (10 mL L^−1^).

Biofertilizers were prepared following the manufacturer’s recommendations. For the EM-1 biofertilizer, 7.8 mL of concentrated EM-1 solution was mixed with 7.8 mL of molasses per liter of autoclaved distilled water, and the mixture was fermented anaerobically for seven days. The PGCM biofertilizer was prepared by dissolving the product at the recommended rate (1 kg of the product per 946 L of water) and aerating it by shaking for 24 h, with molasses added at a rate of one ounce per gallon. The Spectrum biofertilizer was prepared at a concentration of 0.05 g L^−1^, which is higher than the 0.02 g L^−1^ suggested by Soilscape solutions [26], as no colony growth was observed at the lower concentration. We conducted a qualitative assessment of bacterial growth by visual inspection of colonies on PDA rather than employing CFU enumeration on a dedicated bacterial medium. We recognize that this methodology presents a limitation in our study, as it relies solely on visual assessment of PDA to determine bacterial growth. However, our study is based on the recommendations and information declared on the biofertilizers’ labels. Additionally, the Spectrum biofertilizer was supplemented with Pepzyme Clear (SPC) at a rate of 0.3 mL L^−1^ of water. Pepzyme Clear is a liquid biostimulant containing 10% concentrated liquid extracts of non-living soil bacteria, yeast, and fungi cultures in 90% distilled water, used to enhance microbial activation by Soilscape solutions [26]. This biostimulant is commonly used alongside microbial inoculants from Tainio Technology & Technique, Inc.

The commercial biofertilizers evaluated in this study differed substantially in microbial composition, constituent materials, and preparation methods. Given these variations, it is crucial to assess their effectiveness against *B. fagacearum* by considering the overall properties of each biofertilizer.

### 2.3. Antifungal Dual-Culture Plate Assay

Seven-day-old cultures of *B. fagacearum* grown on PDA at 24 °C in darkness were used as inoculum. A 7 mm agar plug of 7-day-old *B. fagacearum* was placed upside-down near the edge of each 90 mmm diameter of PDA plate. On the opposite side (3.5 cm from the fungal plug), a 20 μL droplet of biofertilizer or fungicide solution was deposited and streaked vertically. For the control, 20 μL of autoclaved distilled water was used. The plates were incubated at 24 °C in darkness. Each treatment included six replicates, and the experiment was repeated three times (*n* = 18).

Fresh biofertilizer and control suspensions were prepared for each repetition. For the SPC and PGCM treatments, 10 mL of biofertilizer was placed in a 50 mL Falcon tube with a loose cap to allow aeration. The mixture was then shaken on an orbital shaker (Infors HT Ecotron, Bottmingen, Switzerland) at 220 rpm and 28 °C for 24 h, while the EM-1 solution was utilized after seven days of anaerobic fermentation.

After 15 days, the radial mycelial growth toward the treatment was measured. The percentage inhibition was calculated using the following formula:

(1) $$Inhibition\;growth\;(\%)=\frac{\mathrm{control}\;-\;\mathrm{treatment}}{\mathrm{control}}\times 100$$

where control is the radial growth (cm) in the treated plate with autoclaved distilled water, and treatment is the radial growth (cm) in the treated plate with the biofertilizers or fungicide treatments.

### 2.4. Volatile Organic Compounds Assay

The inhibitory effect of VOCs produced by the commercial biofertilizers on the growth of *B. fagacearum* was evaluated using a modified version of the two-sealed base-plates method described by Gholami et al. [23]. A 20 μL droplet of biofertilizer solution or autoclaved distilled water was streaked vertically at the center of a base PDA plate that did not contain any fungal plug. A second PDA plate was inoculated with a 7 mm fungal plug from a 7-day-old culture, which was positioned near the edge of the plate and inverted over the base plate.

These paired plates were sealed with Parafilm to allow gas exchange of VOCs while preventing physical contact and exchange of non-volatile metabolites [27]. The control plates consisted of fungus paired with autoclaved distilled water. Incubation and replication were performed according to the procedures outlined in Section 2.3. The percentage of inhibition was calculated using the same equation applied in the dual-culture plate assay.

### 2.5. Non-Volatile Metabolites Assay

The SPC and PGCM biofertilizers (10 mL) were cultured separately in loosely capped 50 mL Falcon tubes and incubated in an orbital shaker at 220 rpm and 28 °C for 24 h. The EM-1 solution (10 mL) was prepared seven days in advance and maintained under anaerobic conditions to allow fermentation and activation. After incubation, the resulting cultures were centrifuged at 4500 rpm (4347× *g*) for 20 min at 4 °C (Centrifuge 5910 R, Eppendorf, Hamberg, Germany). The supernatants were filtered aseptically through 0.22 μm sterile membrane filters (Millipore, Burlington, MA, USA) to obtain cell-free filtrates. A 20 μL droplet of cell-free filtrate was added to the corresponding Petri dishes, where a 7 mm fungal plug was inoculated. The dishes were incubated and replicated as described in Section 2.3. After 15 days of incubation, the mycelium radius was measured and the inhibition percentage determined using the formula provided in Section 2.3.

Hyphal morphology was examined in representative plates from each treatment and control using an Axio Scope inverted fluorescence microscope at 100× magnification (Carl Zeiss Axio Observer 7, Jena, Thuringia, Germany). Image processing was performed using Zeiss Zen software, version 3.8. To prepare the samples, a thin section of the growing mycelia was imprinted onto transparent adhesive tape and placed on a drop of blue lactophenol-cotton blue stain (Dawn Scientific Inc., Medford, NJ, USA) on a glass slide. Another drop of lactophenol was added to the tape, followed by a coverslip. The slides were allowed to sit undisturbed for five minutes to enable uniform staining [28].

### 2.6. Statistical Analysis

The data were analyzed using a one-way Analysis of Variance (ANOVA), followed by Tukey’s HSD test to compare the different treatments, using R software version 4.3.3 (v2024, PBC, Boston, MA, USA). To assess homoscedasticity and normality, Levene’s and Shapiro–Wilk tests were conducted. If the assumptions were not met, the Kruskal–Wallis test was employed, followed by the Conover post hoc test with Bonferroni correction. Differences were considered statistically significant at a *p*-value of <0.05.

## 3. Results and Discussion

### 3.1. Antagonism of Commercially Biofertilizers by Dual-Culture Plate Assay

**Figure 1.**
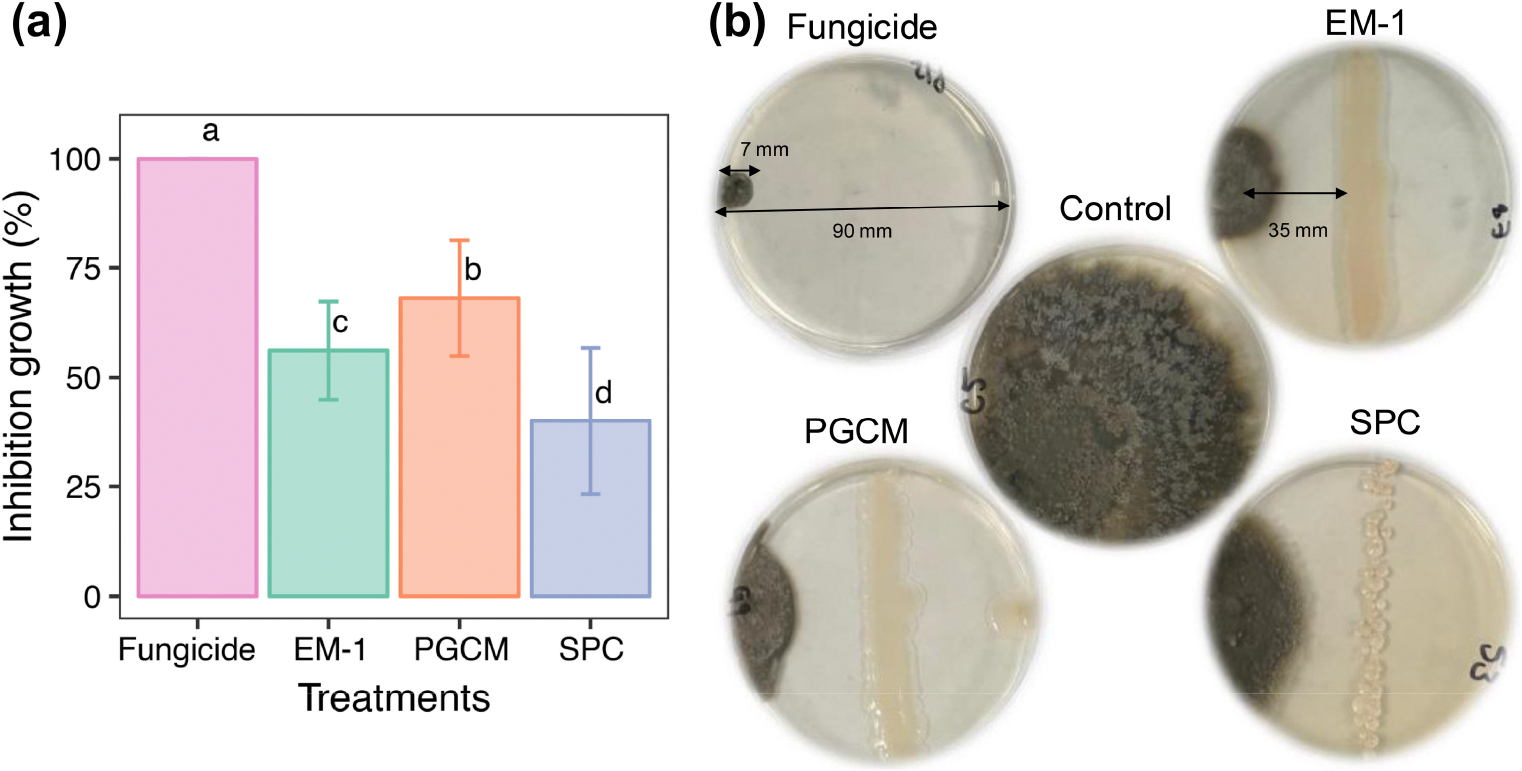
Inhibition of *B. fagacearum* in dual-culture plate assays. (**a**) Percentage of fungal inhibition in the presence of fungicide (Propiconazole 14.3%) and the commercial biofertilizers: Spectrum supplemented with Pepzyme Clear (SPC), EM-1, and Power Gelatinase and Chitinase Microorganism (PGCM), incubated at 24 °C for 15 days, relative to the control (autoclaved distilled water). Error bars represent the standard deviation (*n* = 18). Different lowercase letters indicate significant differences (*p* < 0.05, Conover–Iman’s test) among treatments. (**b**) Representative photographs illustrating fungal growth inhibition from each treatment at the conclusion of the experiment. Labels on the Petri dishes denote original laboratory treatment codes and differ from the treatment abbreviations used in the manuscript. Double arrow lines indicate the scale of the Petri dishes (90 mm), the fungal plug (7 mm), and the scale of the distance from the edge of the fungal plug to the point of commercial fertilizer inoculation (35 mm).

The dual-culture assay provided a quick qualitative assessment of the antagonistic potential of three commercial biofertilizers against *B. fagacearum*. All biofertilizers significantly inhibited mycelial growth compared to the control, although the level of inhibition varied among the treatments (Figure 1). Among the tested biofertilizers, the PGCM treatment demonstrated the strongest antagonistic effect, with a 68% inhibition rate, followed by the EM-1 treatment at 56%. In contrast, the SPC treatment displayed the weakest effect, with only 40% inhibition. The lower effectiveness of the SPC treatment may be due to its relatively low application concentration, as well as suboptimal pH or temperature conditions that could limit microbial activity [29]. Increasing the concentration of the SPC treatment may improve its competitive ability against *B. fagacearum*.

*B. fagacearum* infects the xylem vessels of branches, trunks, and roots, resulting in the production of hyphal masses and mucilaginous gums. These byproducts obstruct the transport of water and nutrients, leading to rapid wilting in red oaks [30]. Our dual-plate assays have demonstrated that treatments using PGCM and EM-1 significantly inhibit the growth of *B. fagacearum*. This might suggest that these microbial communities may outcompete the pathogen for nutrients and space. Additionally, these treatments might facilitate antagonism against the pathogen through a variety of compounds, including enzymes, VOCs, and bacteriocins, as reported for *Bacillus subtilis* [31]. However, these hypothetical mechanisms need further verification. It is important to note that further investigation is necessary to ascertain whether these treatments can reproduce, persist, and successfully colonize xylem tissue in vivo, as this is a critical factor in managing oak wilt disease in field conditions [19].

### 3.2. Effect of Volatile Organic Compounds Produced by Biofertilizers

**Figure 2.**
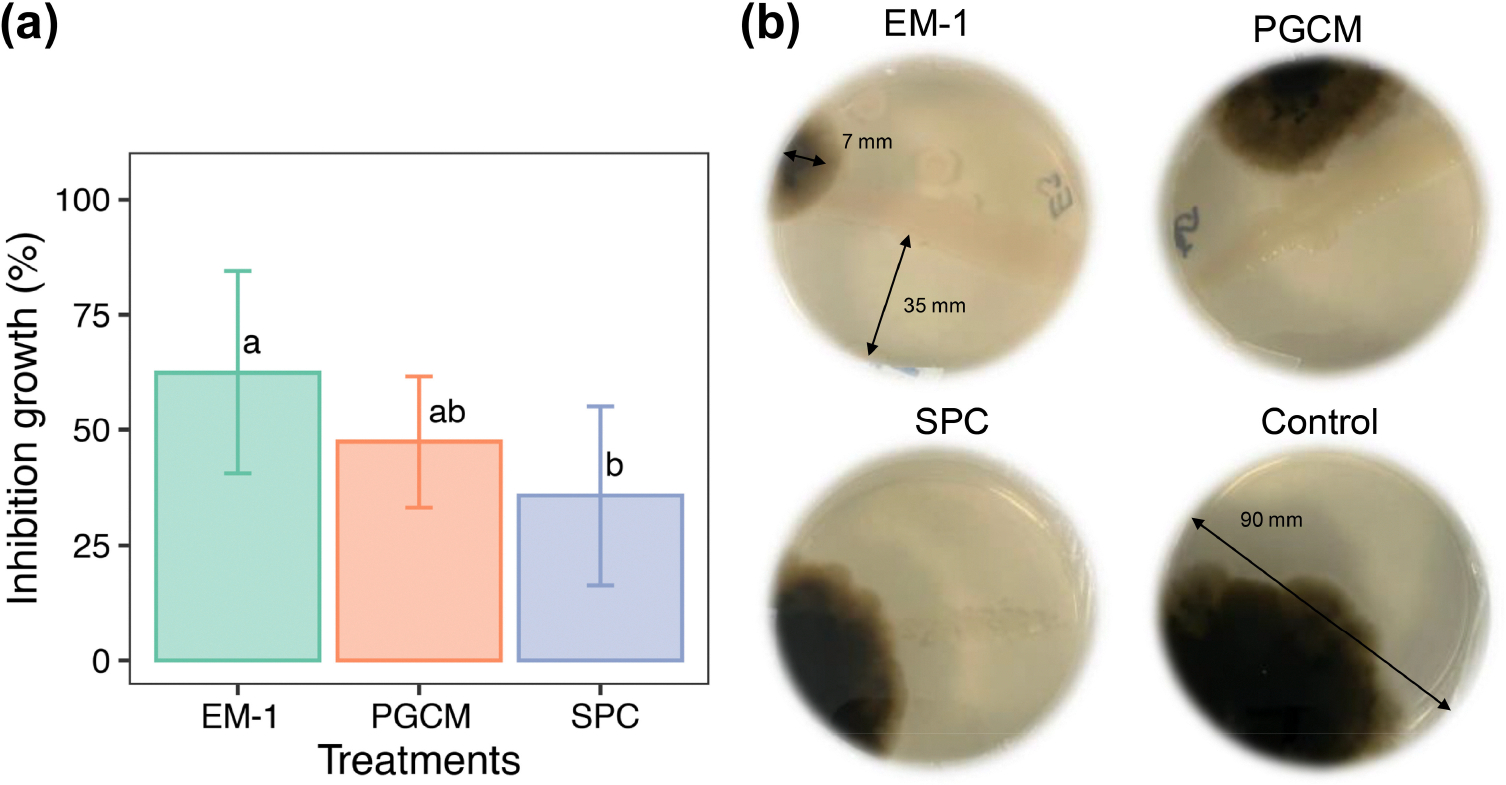
Inhibition of *B. fagacearum* by volatile organic compounds (VOCs). (**a**) Percentage inhibition by VOCs from Spectrum supplemented with Pepzyme Clear (SPC), EM-1, and Power Gelatinase and Chitinase Microorganism (PGCM) after 15 days at 24 °C, relative to the control (autoclaved distilled water). Error bars represent standard deviation (*n* = 18). Different lowercase letters indicate significant differences among treatments (*p* < 0.001, Tukey HSD test). (**b**) Representative photographs illustrating fungal growth inhibition from each treatment at the conclusion of the experiment. Labels on the Petri dishes denote original laboratory treatment codes and differ from the treatment abbreviations used in the manuscript. Double arrow lines indicate the scale of the Petri dishes (90 mm), the fungal plug (7 mm), and the scale of the distance from the edge of the fungal plug to the point of commercial fertilizer inoculation (35 mm).

All biofertilizers produced VOCs that significantly inhibited the mycelial growth of *B. fagacearum* (Figure 2a). However, the biofertilizers varied in their fungal activity. The highest inhibition percentage was observed with the EM-1 and PGCM biofertilizers, at 63% and 47%, respectively, compared to the SPC treatment, which exhibited 36% inhibition. The EM-1 biofertilizer demonstrated a marked ability to inhibit *B. fagacearum* by limiting the growth of typical, high-density hyphae to near the 7 mm fungal plug, with only low-density hyphae visible beyond the inoculation site (Figure 2b).

Similarly, the new mycelia growing from the 7 mm fungal plug displayed a reduction in pigmentation with EM-1 and PGCM biofertilizers compared to the SPC and the control. Previous research has reported that reduced pigmentation in fungal mycelia exposed to VOCs from *Bacillus velezensis* (present in the PGCM biofertilizer) suggests decrease in virulence [32].

This significantly higher efficiency of PGCM and EM-1 relative to the SPC biofertilizer may be attributed not only to their higher concentrations but also to the application of molasses during their activation. Typically, VOCs are associated with microbial growth; however, other studies indicate that adding glucose enhances the production of potent antifungal volatiles by *Bacillus subtilis* [33]. Additionally, the production of VOCs from *Bacillus velezensis* might be influenced by the growth medium and its metabolic capabilities [34].

These findings imply that VOCs may possess antifungal properties, as these compounds alter hyphal development and pigmentation, potentially diminishing the infectious capacity of *B. fagacearum*. However, this effect appears to depend on the specific strain [35].

*B. fagacearum* emits a VOC profile in culture 24–48 h post-inoculation, even before the onset of visible mycelial growth [36]. These VOCs release a fruity odor in infected trees, attracting nitidulid beetles that vector the fungus and facilitate its above-ground spread among injured trees in the field [12,37]. Our VOC assays indicated that PGCM and EM-1 treatments reduced *B. fagacearum* growth in a shared headspace. This observation might suggest that the microbial volatiles produced by these treatments may function as biofumigants, potentially inhibiting the pathogen’s metabolic activity or altering its volatile emissions. However, further analysis of the VOC profile will be necessary to determine which specific VOCs from PGCM and EM-1 inhibit fungal growth.

### 3.3. Effect of Non-Volatile Metabolites and Hyphal Morphology

**Figure 3.**
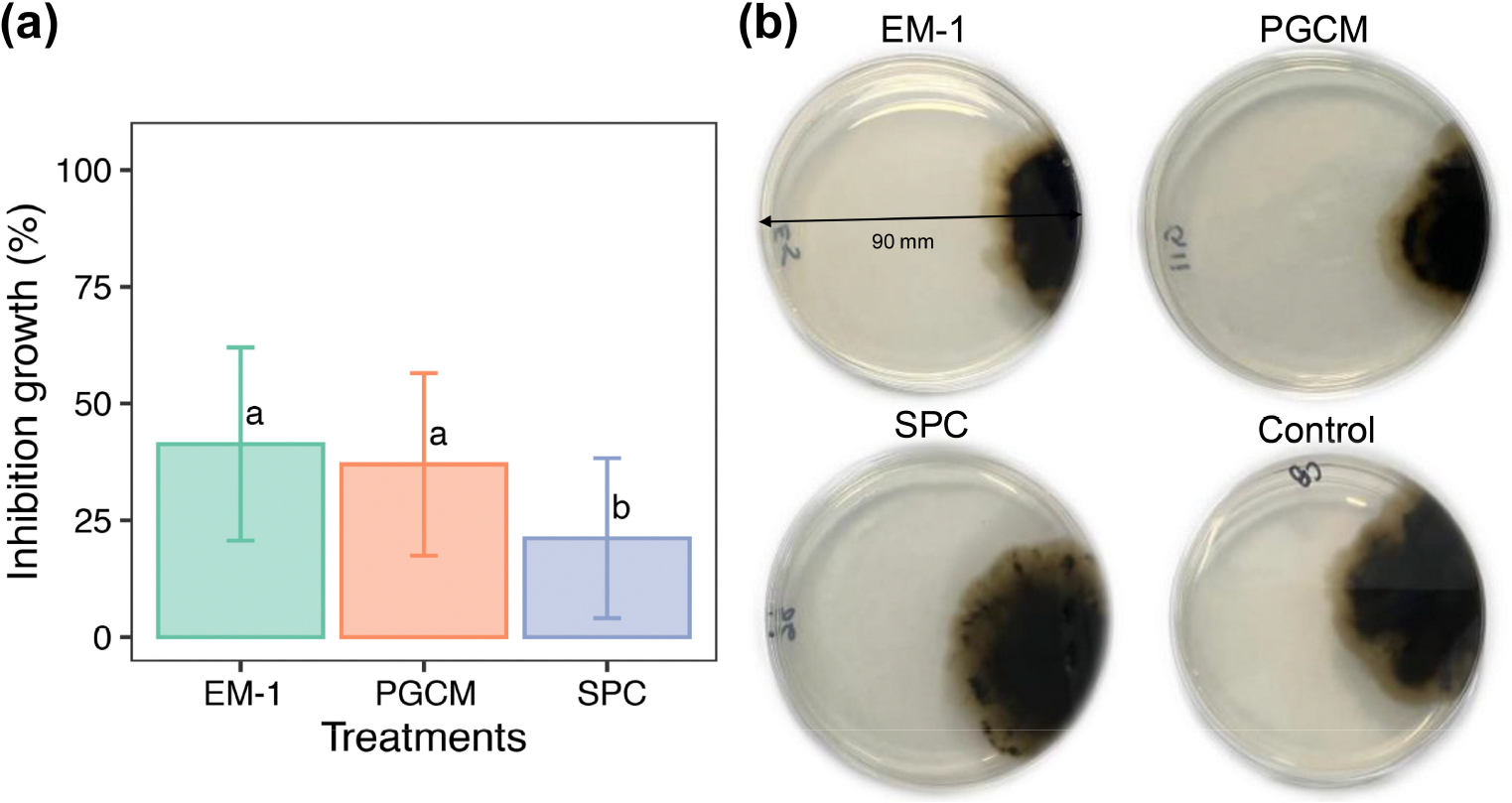
Inhibition of *B. fagacearum* by non-volatile metabolites. (**a**) Percentage inhibition by cell-free filtrates from Spectrum supplemented with Pepzyme Clear (SPC), EM-1, and Power Gelatinase and Chitinase Microorganism (PGCM) after 15 days at 24 °C, relative to the control (autoclaved distilled water). Error bars represent the standard deviation (*n* = 18). Different lowercase letters indicate significant differences among treatments (*p* < 0.01, Tukey HSD test). (**b**) Representative photographs illustrating fungal growth inhibition from each treatment at the conclusion of the experiment. Labels on the Petri dishes denote original laboratory treatment codes and differ from the treatment abbreviations used in the manuscript. Double arrow line indicates the scale of the Petri dishes (90 mm). A 7 mm fungal plug was placed upside-down near the edge of each Petri dish. On the opposite side, 35 mm from the fungal plug, a droplet of non-volatile metabolite solution was streaked vertically.

Cell-free filtrates from all biofertilizers inhibited mycelial growth, although the effect varied among biofertilizers (Figure 3). The EM-1 and PGCM treatments showed the highest inhibition percentages at 41% and 37%, respectively. In contrast, the SPC treatment exhibited the lowest inhibition rate at 21%. Overall, the non-volatile metabolites produced by all the biofertilizers demonstrated weaker antifungal effects than the dual-culture plate and VOCs assays, which aligns with previous studies on non-volatile metabolites [23]. This finding aligns with a study on *Lactobacillus paracasei*, which indicated that antifungal activity might be underestimated when a cell-free culture is used rather than an assay that analyzes VOCs [38]. The underestimation occurs due to cell loss during centrifugation and infiltration. These steps were used in this study to obtain a cell-free culture for analyzing only the effects of non-volatile metabolites.

**Figure 4.**
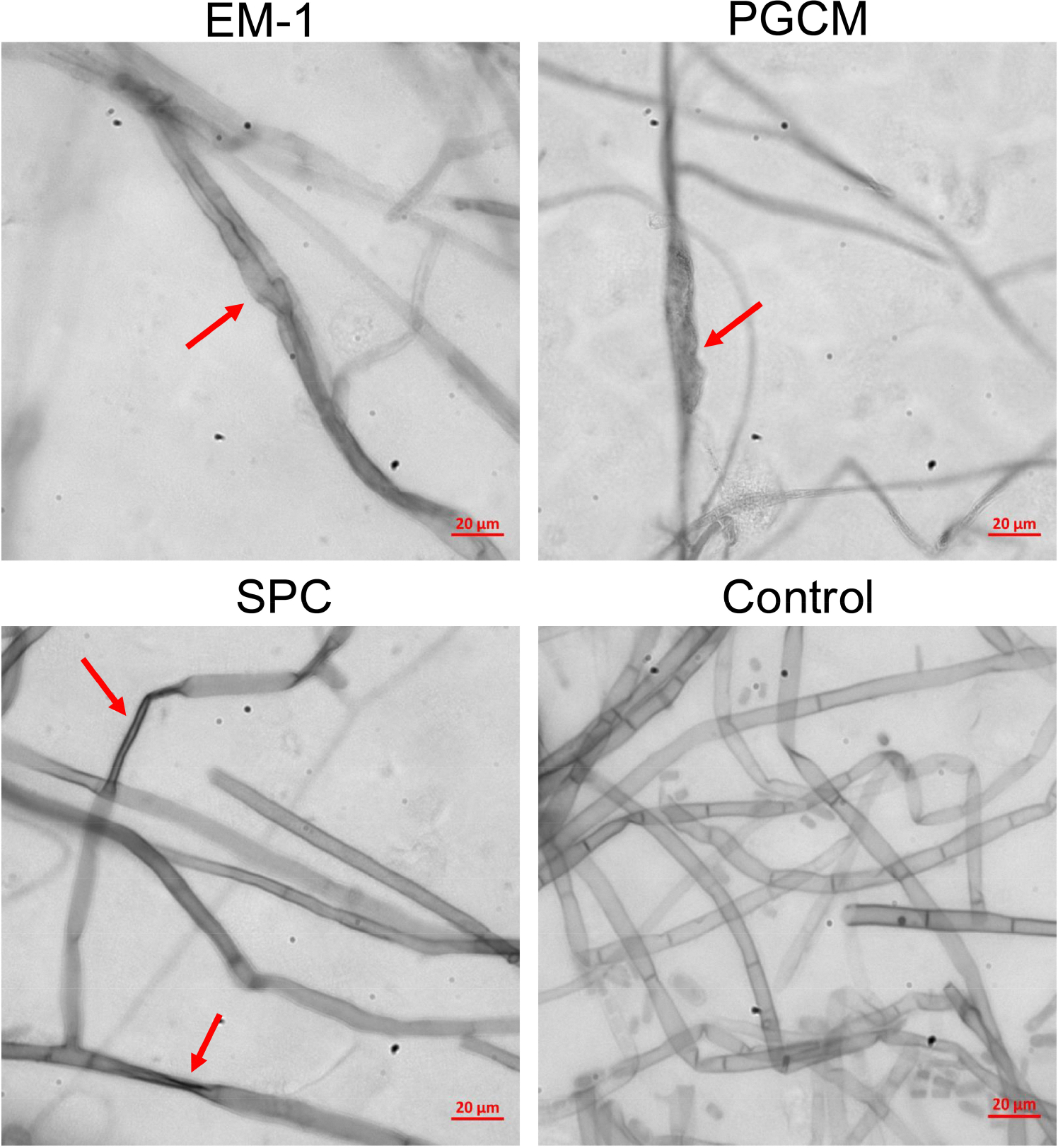
Hyphal morphology of *B. fagacearum* analyzed after exposure to non-volatile metabolites at 24 °C for 15 days from EM-1, Power Gelatinase and Chitinase Microorganism (PGCM), Spectrum supplemented with Pepzyme Clear (SPC), and control (autoclaved distilled water). Red arrows indicate severe distortion and swelling in the EM-1 and PGCM treatments, while constrictions were observed in the SPC treatment, also marked with red arrows.

Microscopic examination revealed significant changes in the hyphal morphology of *B. fagacearum* after exposure to non-volatile metabolites from biofertilizers, in comparison to the control (Figure 4). The control hyphae were uniform, smooth, and homogeneous, with cylindrical principal axes and intact surfaces. In contrast, hyphae exposed to filtrates from the EM-1 and PGCM treatments showed severe damage, including loss of structural integrity, distortion, irregular swelling, and bulbous formation. The SPC treatment produced milder effects, including constriction, thinning, and a reduction in hyphal diameter in certain sections.

The mycelial growth inhibition observed in the dual-culture plate and non-volatile metabolite assays may be attributed to the extracellular lytic enzymes (e.g., protease, glucanase, cellulase, gelatinase, lipase, amylase, chitinase) and siderophores produced by the biofertilizers. This information is provided by the manufacturer for the PGCM biofertilizer. Chitinase, glucanase, and protease are known to degrade fungal cell wall components, resulting in hyphal swelling or the appearance of bulbous or shrunk hyphae, which suppresses fungal growth [23,28]. Therefore, chitinase and glucanase activities may be one of the significant antagonistic mechanisms of biocontrol in our tests with PGCM, as fungal cell walls include polysaccharides, such as chitin and glucan [39]. However, further research is needed to verify whether these enzymes have contributed to cell wall degradation, leading to hyphal alteration in *B. fagacearum*. Additionally, *Bacillus* species such as *Bacillus subtilis* and *Bacillus velezensis*, which are present in the EM-1 and PGCM biofertilizers, respectively, are known to produce a variety of non-volatile lipopeptides with antifungal properties. The production of these lipopeptides is influenced by factors such as agar medium, culture conditions, carbon and nitrogen sources, trace metals, temperature, incubation time, concentration, and agitation speed [40]. Consequently, the more potent inhibitory effects of the EM-1 and PGCM biofertilizers compared to the SPC may indicate a higher activity or production of these antifungal metabolites under the experimental conditions.

The commercial biofertilizers evaluated in this study differed substantially in microbial composition, constituent materials, and preparation methods, which may have influenced their efficacy against *B. fagacearum* and limited strict head-to-head biological comparisons among treatments. Nevertheless, all products were prepared according to current commercial recommendations to reflect typical farming practices better. Therefore, the observed effects of the biofertilizers likely resulted from interactions among their constituents, microbial communities, and preparation methods, rather than from any single component or microorganism alone. Additionally, biofertilizers were used as commercially formulated and labeled, without independently analyzing their complete microbial composition or verifying all listed ingredients. This approach reflects the practical use of proprietary commercial products and the limited quality control associated with product labeling, which often makes it difficult for growers to distinguish between products with demonstrated efficacy and those without [41]. Consequently, this restricts our ability to attribute specific effects to individual components. Future research should focus on further characterization and dose–response studies of these biofertilizers.

The preliminary experiments described here were conducted in vitro. When these commercial fertilizers are applied in the field, they must compete with other soil microorganisms and withstand environmental conditions that could affect their efficacy as biocontrol agents. Additionally, it is essential that they can effectively colonize the xylem [19]. Consequently, further field studies are required to validate their antagonistic effects against *B. fagacearum*.

## 4. Conclusions

This study demonstrates that the EM-1 and PGCM biofertilizers exhibited antifungal activity against *B. fagacearum* in vitro. The dual plate assay showed inhibition of mycelial growth, while the production of VOCs and non-volatile metabolites by both EM-1 and PGCM also contributed to this inhibition and led to notable alterations in hyphal structure. Given the observed effects on fungal growth and morphology, it is probable that a diverse array of compounds is involved. Consequently, future research is needed to identify these VOCs and non-volatile metabolites.

PGCM and EM-1 might be promising options for further investigation to enhance current management strategies, which often exhibit variable efficacy and lack biological approaches. Additionally, since these BCAs are already available as commercial biofertilizers for enhancing nutrient levels, farmers may find them easy to adopt and handle when used according to the provided guidelines.

Future research should evaluate these biofertilizers under field conditions to determine their effectiveness on diseased trees, which environmental factors and soil–tree–microbe interactions can influence. Key factors to investigate include seasonal variations in nutrient availability within the vascular system, the optimal timing and methods of application (e.g., trunk injection), and the distribution of microorganisms within the tree. Each of these factors may influence the biofertilizers’ inhibitory effectiveness against *B. fagacearum* and their adaptation after introduction into the vascular systems of oak trees. Such studies will be crucial for translating the in vitro potential of these biofertilizers into practical disease management strategies.

## Supplementary Material

SI materials

**Supplementary Materials:** The following supporting information can be downloaded at: https://www.mdpi.com/article/10.3390/microbiolres17060115/s1. The supplementary material includes raw data of dual plate assay, volatile organic compounds, and non-volatile organic compounds. Table S1 Percentage growth inhibition of *Bretziella fagacearum* in each Petri dish during the dual plate assay conducted with different commercial biofertilizers. Table S2 Percentage growth inhibition of *Bretziella fagacearum* in Petri dishes during the volatile organic compounds assay conducted with different commercial biofertilizers. Table S3 Percentage growth inhibition of *Bretziella fagacearum* in Petri dishes during the non-volatile organic compounds assay conducted with different commercial biofertilizers.

## Data Availability

The original contributions presented in this study are included in the article/Supplementary Material. Further inquiries can be directed to the corresponding author.

## Abbreviations

The following abbreviations are used in this manuscript:

BCAs: Biological control agents
PGCM: Power Gelatinase and Chitinase producing Microorganism
PDA: Potato Dextrose Agar
VOCs: Volatile organic compounds
